# A Case of Rectovesical Fistula Following Blunt Trauma in a Child

**DOI:** 10.7759/cureus.18931

**Published:** 2021-10-20

**Authors:** Yadavalli R D Rajan, Chandana Priyanka

**Affiliations:** 1 General Surgery, Siddhartha Medical College, Vijayawada, IND

**Keywords:** rectovesical fistula, urinary diversion, fecal diversion, perineal trauma, blunt trauma

## Abstract

Rectovesical fistulae (RVF) are uncommon entities and usually occur after surgery for prostate, radiation, and sometimes due to penetrating trauma. However, RVF occurrence after blunt trauma to the abdomen or perineum is very rare. The management of RVF is challenging, and treatment options should be considered according to the individual. Here we present a case of a 10-year-old boy who presented with fecaluria, pneumaturia, the passage of urine per rectum, and burning micturition for four days after incurring a blunt injury to the perineum. Cystography revealed leakage of contrast material into the rectum and an MRI of the pelvis was done for confirmation, which revealed a 1.3 cm thick fistulous tract of 2.7 cm length with openings at the posterior bladder wall and anterior rectal wall. After conservative management for 14 days failed to show any improvement, primary repair of the fistulous tract along with fecal diversion and urinary diversion were done. The suprapubic catheter was removed after four weeks, and at the two-month follow-up, colostomy closure was done. No recurrence was found in the six-month follow-up period. In cases of small traumatic RVF where conservative management fails, fecal and urinary diversion can be considered, as it is associated with successful outcomes and less recurrence.

## Introduction

Rectovesical fistulae (RVF) are uncommon entities. Common causes include prostatectomy for cancer of the prostate or benign prostatic hyperplasia and radiation. Rectovesical fistulae due to trauma are not so common and could be caused by gunshot injury, foreign bodies inserted in the rectum, road traffic accidents, and rarely due to blunt trauma. They usually present with pneumaturia and fecaluria, and detecting the exact location of rectovesical fistulae needs the aid of diagnostic imaging. The management of RVF is challenging and varies according to the etiology, and nearly half of them heal by urinary and fecal diversion while few patients need definitive procedures. After a thorough literature search, we could say that RVF due to blunt trauma in the pediatric population is very rare, and only a few such cases have been reported so far.

## Case presentation

**Figure 1:**
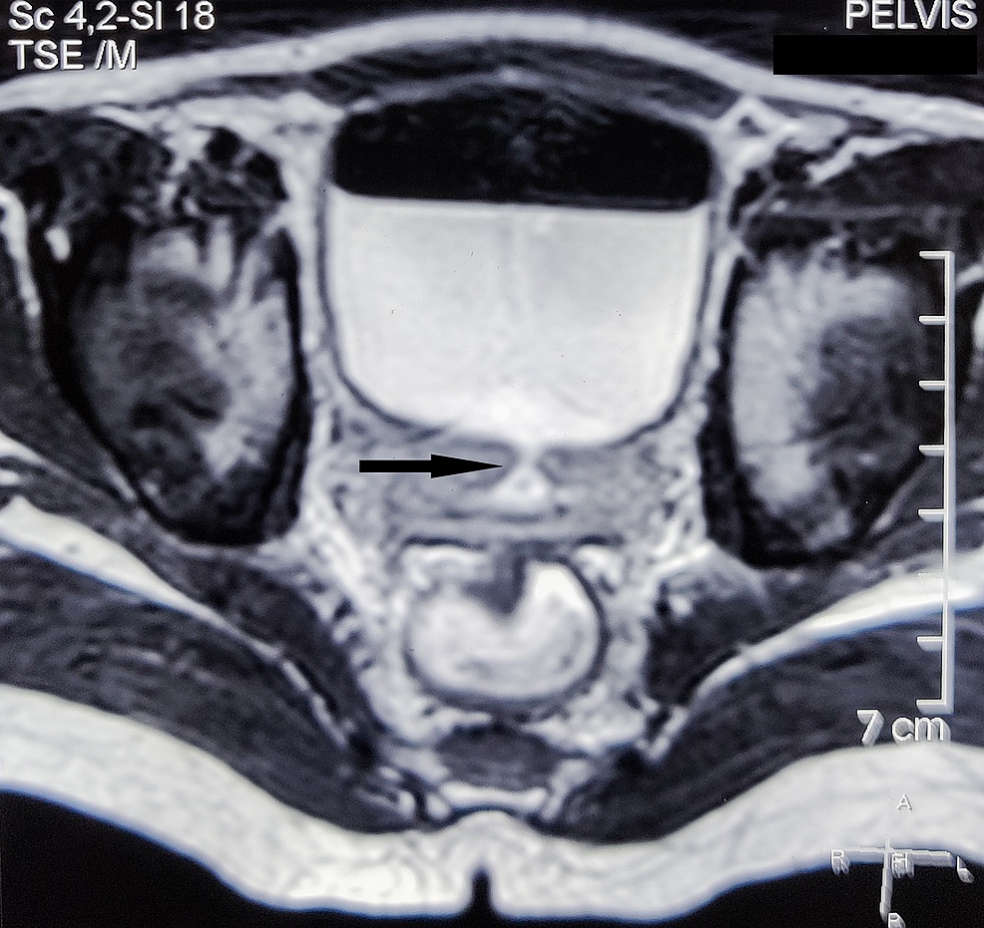
Axial section of MRI of the pelvis showing the fistulous tract (arrow)

**Figure 2:**
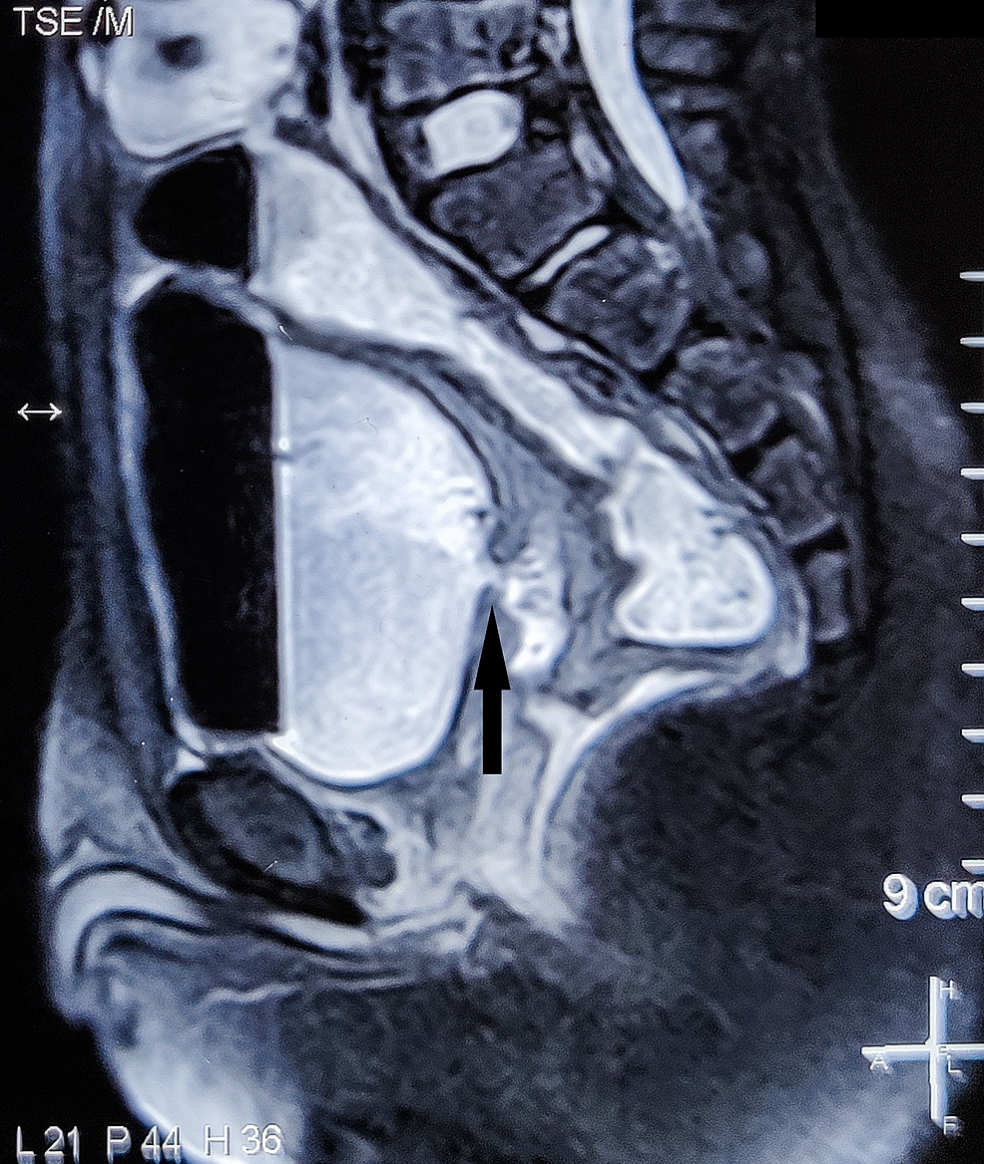
Saggital section of MRI of the pelvis showing the fistulous tract (arrow)

A 10-year-old boy presented with fecaluria, pneumaturia, the passage of urine per rectum, and burning micturition. The patient revealed a history of trauma to the perineal region while riding a bicycle two days before the onset of symptoms, following which he developed perineal and suprapubic discomfort. Digital rectal examination showed no blood in the rectum, but a defect was palpated in the anterior rectal wall. Laboratory examination revealed leukocytosis (16,000 cells/cu. mm). Urine analysis showed fecal contamination, 10-15 pus cells/hpf, and granular casts. Urine culture revealed a growth of Escherichia (E.) coli > 10^5^cfu/mL. Cystography was done, which revealed the leakage of contrast material into the rectum, thus substantiating the presence of a rectovesical fistula. MRI of the pelvis was done for better visualization of the fistulous tract and surrounding tissues, which revealed a 1.3 cm thick fistulous tract of 2.7 cm length with openings at the posterior bladder wall and anterior rectal wall as shown in Figures 1-2.

**Figure 3:**
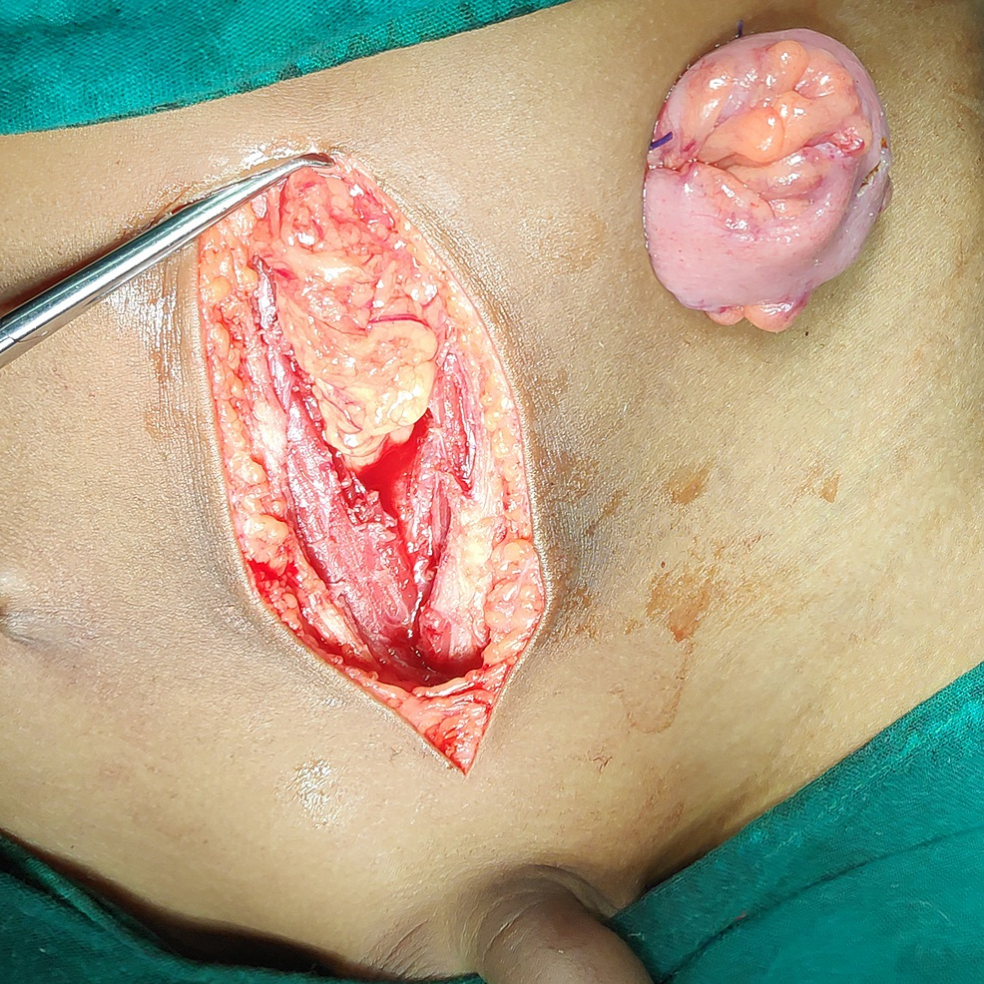
Intraoperative picture showing colostomy An intraoperative image of the fistulous tract could not be obtained.

The patient was initially treated conservatively with antibiotics, urethral catheterization, and bowel rest for 14 days but showed no resolution. Mechanical bowel preparation was done, and IV antibiotics were given preoperatively. Under general anesthesia, a midline infra-umbilical incision was made, and the posterior aspect of the bladder and the anterior rectal wall was visualized. No necrotic or inflammatory tissues were seen surrounding the fistula. A longitudinal cystotomy was done, and a small defect on the posterior bladder wall was identified and repaired in a single layer. Cystotomy was closed with a suprapubic catheter in situ for urinary diversion. End sigmoid colostomy with mucus fistula was kept for fecal diversion as shown in Figure 3. After giving a course of IV antibiotics for five days, the patient was discharged after the removal of the Foley catheter. The suprapubic catheter was removed after four weeks, and at the two-month follow-up, colostomy closure was done. No recurrence was found during the six-months follow-up period.

## Discussion

Rectovesical fistulas are very rare and can be caused by congenital anorectal malformations, bladder and prostate surgery, gynecologic and colorectal surgery, radiotherapy for pelvic tumors, trauma, and neglected foreign bodies [1]. Pneumaturia is the most common and pathognomonic symptom. Fecaluria and passage of urine per rectum are the next most common symptoms [2]. A rectovesical fistula can cause many complications if left untreated, including recurrent urinary tract infections, pelvic abscesses, and peritonitis [3]. Cystography, endoscopy, computed tomography (CT), and magnetic resonance imaging (MRI) are diagnostic investigations that aid in the diagnosis of RVF. Ultrasonography is also sensitive in detecting RVF, but it fails to define the complexity of the fistula. CT is highly sensitive in detecting RVF, and it has the advantage of detecting the extra-luminal disease process pertaining to the formation of the fistula. The sensitivity and specificity of MRI in diagnosing RVF is nearly 100%. Intravenous gadolinium usage substantially improves the detection of these fistulae [2]. T1-weighted images provide delineation of the extension of the fistula relative to adjacent bowels and show inflammatory changes. T2-weighted images show fluid collections within the fistula and localized fluid collections in the extraintestinal tissues [4].

Conservative management of RVF includes bowel rest, urethral catheterization, antibiotics, and a short course of low-dose steroids. This line of management is reserved for minimally symptomatic fistulas that are not associated with malignancy. Surgery is the mainstay of treatment for most RVF. Various endoscopic, laparoscopic, and open surgeries have been depicted in the literature. Historically, primary colostomy for fecal diversion has been used for treating these fistulae [5]. Open surgery consists of one-staged or multiple-staged procedures, including resection of bowel and anastomosis, repair of the bladder, colostomy, and colostomy closure at a later stage. Some studies showed that one-staged surgery with primary resection and anastomosis without diverting stoma could be done in the majority of cases [2]. Bladder repair needs to be done in large visible defects by either oversewing or excision and will not affect the outcome. Intraoperatively, methylene blue dye can be injected per-urethra to detect small defects in the bladder wall. Small defects that are not conspicuous need not be repaired and can be left to heal spontaneously [4]. Considering the omentum's rich vascularity and immunological properties, some authors have described the interposition of an omental flap between the bladder and the bowel, as it is believed to aid in the healing of fistulas and reduction of recurrence rates [2].

Thurairaja et al. described conservative management of RVF in which a 46-year-old man sustained an injury to the rectum after falling onto a chair. The patient was kept on antibiotics for three weeks, and a suprapubic catheter was kept in situ for five weeks, after which a CT scan revealed no air in the bladder [6]. Sotelo et al. described both laparoscopic and robot-assisted techniques in the repair of RVF [7-8]. Their technique included laparoscopic cystotomy and dissection of the fistulous tract, closure of the rectum, the interposition of the omentum between the bladder and rectum, and closure of the bladder, followed by suprapubic cystostomy and colostomy. Although this technique was successful, it is technically demanding. Todd et al., in a series of 24 patients, with recto-urinary fistulas, and David et al., in a series of 51 patients with recto-urinary fistulas, used the York-Mason posterior, transanal, and transrectal approach to the repair of recto-urinary fistulas and have shown 91.6% [9] and 92% [10] success rates, respectively, and that diversion colostomies can be avoided. Walker et al. described a successful transvesical technique for the repair of a traumatic rectovesical fistula in a 14-year old boy. Primary repair of the bladder defect was performed, and diversion colostomy and suprapubic catheterization were done to divert feces and urine, respectively. Colostomy closure was completed at a later stage [11].

Incidence of RVF due to blunt injury to the perineum is very rare and only a few such cases have been reported in the pediatric population. Although many techniques have been described in the literature, no standard treatment technique has been established and the choice of technique differs based on the individual. In our case, we used a transabdominal approach to visualize both the bladder and the rectum. Cystotomy was done and the posterior bladder wall was repaired, following which the cystotomy was closed. Given the small diameter of the fistula and because the surrounding tissues were not inflamed, primary repair of the defect on the posterior bladder wall was done without excision of the fistulous tract or repair of the anterior rectal wall. Diversion colostomy with mucous fistula was done for fecal diversion. Suprapubic catheter and foley's catheter per urethra were used for urinary diversion. This technique is not technically demanding and can be performed easily compared to transanal and transrectal approaches. The transabdominal technique also provides clear visualization when both the rectum and the bladder need to be repaired.

## Conclusions

Although multiple techniques have been described in the literature for the operative treatment of RVF, no standard technique has been established. In small traumatic fistulas, particularly in children, transabdominal primary repair of the bladder defect along with urinary and fecal diversion can be done with successful outcomes and has less recurrence.
